# Developing and piloting a set of quality-of-care indicators for Romanian public hospitals as part of a national programme to fund quality

**DOI:** 10.1186/s12913-024-11462-6

**Published:** 2024-10-16

**Authors:** Damir Ivanković, Válter R. Fonseca, Angeliki Katsapi, Angeliki Karaiskou, Georgios Angelopoulos, Dragos Garofil, Alexandru Rogobete, Niek Klazinga, Natasha Azzopardi-Muscat, João Breda

**Affiliations:** 1 WHO Office on Quality of Care and Patient Safety, WHO Regional Office for Europe, Ploutarchou 3, Athens, 10675 Greece; 2 Carol Davila University of Medicine and Pharmacy, Bucharest, Romania; 3 Victor Babes University of Medicine and Pharmacy, Timisoara, Romania; 4 Public and Occupational Health, Amsterdam UMC Location University of Amsterdam, Amsterdam, Kingdom of the Netherlands; 5 Quality of Care, Amsterdam Public Health research institute, Amsterdam, Kingdom of the Netherlands; 6 Division of Country Health Policies and Systems, WHO Regional Office for Europe, Copenhagen, Denmark

**Keywords:** Quality of Health Care, Quality Improvement, Quality indicators, Hospitals, Romania

## Abstract

**Background:**

Healthcare systems aim to enhance the health status and well-being of the individuals and populations they serve. To achieve this, measuring and evaluating the quality and safety of services provided and the outcomes achieved is essential. Like other countries, Romania faces challenges regarding the quality of care provided in its public hospitals. To address this, the Romanian Ministry of Health initiated reforms in 2022, including implementing a pay-for-performance model based on quality indicators. This paper presents a descriptive analysis of processes, methods, results and lessons learned from developing and piloting a set of Quality of Care indicators for Romanian public hospitals.

**Methods:**

World Health Organization’s Athens Office on Quality of Care and Patient Safety assisted Romania in developing and piloting a set of quality-of-care indicators for public hospitals. The development phase included defining indicator domains, identifying potential indicators across these domains, and defining the final indicator set. The piloting phase involved selecting and recruiting piloting hospitals, developing data collection and validation methods and tools, training hospital staff, and collecting and analysing indicator data. Piloting ended with an evaluation workshop. Mixed, quantitative and qualitative methods were used, including literature reviews, stakeholder consultation workshops, survey instruments developed for this study, modified Delphi panels and consensus-building meetings. National stakeholders were actively involved throughout the process.

**Results:**

Four priority domains were defined for quality-of-care indicators for Romanian public hospitals: patient safety, patient experience, healthcare workforce training and safety, and clinical effectiveness. 25 core indicators were selected across these domains. During the pilot, hospitals achieved an average completion rate of 90% for data submission, with all domains rated equally relevant during post-pilot evaluations. Lessons included the need for supportive legislation, improved internal auditing practices and enhanced staff training, refinement of indicator data collection methods and alignment of indicators with hospital-specific contexts.

**Conclusions:**

This work presents a significant stride in improving Romanian public hospitals’ quality of care and patient safety. It underscores the importance of high-level commitment, stakeholder engagement, and robust data practices in driving successful quality improvement efforts. Emphasising the role of data-driven and patient-centric approaches in achieving optimal healthcare outcomes, lessons learned offer insights for the continuation of quality improvement work in Romania but also for healthcare systems elsewhere.

**Supplementary Information:**

The online version contains supplementary material available at 10.1186/s12913-024-11462-6.

## Introduction

The main aim of healthcare systems is to improve the health status and well-being of individuals and the populations they serve [1]. This is achieved by effectively delivering high-quality, value-based healthcare services and efficiently utilising available resources [2]. By providing evidence-based, effective, safe, people-centred, timely, equitable, integrated, and efficient services, healthcare systems can fulfil the expectations of the patients, families, caregivers, and health workforce. To increase the likelihood of desired health outcomes, health systems and their facilities must be able to measure and evaluate the quality of care (QoC) provided [3, 4].

The Romanian healthcare system consists of both public and private providers aiming to provide universal health coverage (UHC). However, it faces challenges such as underfunding, resource shortages, and uneven quality and access to care. Romania has some of the highest treatable and preventable mortality rates in the European Union (EU), while the self-reported unmet needs are double the EU average. Additionally, the migration of healthcare professionals to other countries has led to shortages in the health workforce. In 2021, per capita spending on health in Romania was 1.663 EUR, less than half the EU average, while health expenditure made up for 6.5% of gross domestic product (GDP). Despite a recent drop in GDP, public spending on health continues to grow [5, 6].

The health system in Romania is hospital-centric, with a total of 567 hospital facilities, two-thirds of which (366) are public ones and are owned and managed by the Ministry of Health (MoH) and local authorities [7]. The system is characterised by unsustainably high spending on hospitals and overutilisation of hospital services without a relevant positive impact on health outcomes. This unbalanced use of services has led to strained hospital infrastructure and concerns over patient safety, including almost a three-fold rise in rates of healthcare-associated infections (HAIs) between 2012 and 2020, particularly in public hospitals. Additionally, issues of severe underreporting of HAIs have been widely acknowledged, with legislative and organisational initiatives underway to tackle those challenges [8–10]. Urban areas generally offer more advanced and specialised services, resulting in better outcomes compared to rural counterparts. Long wait times for procedures and consultations and insufficient access to state-of-the-art diagnostics and treatments contribute to delayed diagnosis and treatment, patient dissatisfaction and suboptimal outcomes [11].

As presented in this paper, despite and due to these challenges, initiatives to improve care delivery and patient experience, modernise facilities, and strengthen workforce capacity are underway. These efforts focus on improving the quality and safety of care provided across the country’s existing public hospital network and, consequently, health outcomes.

In 2021, the European Commission (EC) adopted a positive assessment of Romania’s post-COVID-19 Recovery and Resilience Plan (RRP), financially supporting the implementation of crucial national reforms. The RRP’s Healthcare Services component focuses on improving the Romanian healthcare system’s management, accountability and resilience [12]. As part of this reform, in 2022, the Ministry of Health asked the WHO Regional Office for Europe for support in setting up a Health Quality Fund (HQF), a monetary incentive system for public hospitals that aims to improve QoC and patient outcomes in Romanian public hospitals. WHO Office on Quality of Care and Patient Safety was mandated to support, develop and implement a set of hospital QoC indicators to aid the national rollout of HQF [13].

This paper presents a descriptive analysis of processes, methods, results and lessons learned from developing and piloting a set of QoC indicators for Romanian public hospitals.

## Methods

In 2022 and 2023, a multi-phase mixed-methods approach was used to develop and pilot a set of QoC indicators for public hospitals in Romania [14–16]. WHO Office on Quality of Care and Patient Safety undertook this work to assist the Romanian MoH in setting up a national HQF and incentivising QoC improvement in public hospitals. The authors are public health researchers and consultants in health systems and services, QoC, and patient safety and were actively involved in technical support activities described here.

The methodology focused on developing a national hospital set of QoC indicators, piloting them in a sample of Romanian public hospitals and distilling lessons learned for the subsequent technical work. This study employs a descriptive research design, which is particularly suited for documenting and analysing complex health system interventions. Our approach allows for a detailed exploration of the development and implementation process of QoC indicators in a real-world setting, providing valuable insights for future quality improvement initiatives in similar contexts. Indicator set development involved three steps: defining indicator set domains, identifying potentially applicable indicators per each domain and developing the final set of QoC indicators alongside their methodological considerations. Aiming to evaluate the feasibility of indicator data collection and inform subsequent project phases, the QoC indicator set piloting also involved multiple steps [17, 18]. It included identifying piloting hospitals, developing a comprehensive methodology for data collection with inclusion criteria and validity control, setting up an information technology (IT) data collection platform, training piloting study participants in data collection, and indicator work in general. Following the piloting study data collection and analysis, a post-piloting technical workshop was organised to present initial results and elicit participant feedback. Table 1 presents an overview of the project phases, steps and methods. These are also described in more detail in the following paragraphs.

**Table 1:** An overview of phases, steps and methods of the development and piloting of a set of quality and safety indicators in Romanian public hospitals as part of a national programme for funding QoC (2022–2023)

Phases	Steps	Methods	Timeframes	Outputs
QoC indicator set development	Definition of indicator set domains	Desk research and stakeholder consultations- based environmental scan	2022 Q3	A list of key indicator domains for QoC improvement work in Romanian hospitals
	Identification of potential indicators	Literature review	2022 Q4	A broad list of potential QoC indicators per defined domains
	Development of the set of indicators	Modified Delphi consensus-building processes and technical work	2022 Q4–2023 Q1	Core set of QoC indicators, including detailed methodological definitions
QoC indicator set piloting	Selection of piloting hospitals	Criteria development and stakeholder consultations	2023 Q1–2023 Q2	A list of public hospitals in Romania to participate in the piloting
	Development of data collection tools and validation methods	Desk research, literature review and stakeholder consultation	2023 Q2	Technical guidebook for the piloting process, defining data measurement and collection considerations, indicator quality and validity criteria and clinical assessment validation processes
	Development and implementation of the data collection IT platform	Technical requirements definition, IT solution provider sourcing and platform deployment	2023 Q2	An IT data collection and sharing platform for the piloting of the core set of QoC indicators in the piloting hospitals
	Training of staff in participating pilot hospitals	On-site training	2023 Q2	Piloting hospitals’ participating staff trained in QoC indicator set data collection and sharing
	Pilot data collection and analysis	Data collection through the IT platform and statistical analysis	2023 Q3	QoC indicator data collected and analysed from piloting hospitals
	Piloting phase evaluation	Technical workshop with participants and stakeholders, including a survey instrument developed for this study	2023 Q4	Collection of experiences and recommendations from the QoC indicator data piloting phase

Finally, the authors reflected on the technical assistance work of developing and piloting the QoC indicator set in Romanian public hospitals in 2022 and 2023, and identified lessons learned to support the next steps of the project.

### QoC indicator set development

The development of the QoC indicator set for Romanian public hospitals started with identifying broad indicator domains and was based on desk research and stakeholder consultations. Preparatory desk research focused on familiarisation with available health system assessment data for Romania and reviewing best national practices in defining and implementing a national set of hospital QoC indicators. This methodological step was based on environmental scan methodology with an aim of identifying indicator domains most relevant to the Romanian healthcare context and used internal and external person and non-person sources [19]. Person sources included national stakeholders and international experts, such as health system administrators and managers working with the Romanian MoH, National Health Insurance House (NHIH), National Authority of Quality Management in Health (ANMCS), as well as hospital managers and administrators, health systems and services researchers and front-line workers, including those affiliated to the Romanian College of Physicians (CMR) and the Order of Nurses, Midwives and Medical Assistants in Romania (OAMR). Non-person sources included both publicly available and internal reports assessing various quality and safety aspects of the Romanian healthcare system. Examples included internal policy documents as well as publicly-available international (comparative) reports, such as those published by the WHO, EU, Eurostat, Organisation for Economic Co-operation and Development (OECD), World Bank and others. The results of these reviews were used as input for country missions and further stakeholder engagement. In-country consultations involved a broad and continuous stakeholder engagement. The definition of indicator set domains was focused on identifying key needs and priorities for Romania’s QoC and patient safety improvement efforts. Next, an extensive set of potential hospital QoC indicators was identified, based on available research- and practice-based literature, across all domains [16, 20, 21]. Again, an environmental scan methodology-based approach was used to screen relevant indicators and include them in the in preliminary list of potential QoC indicators to be used in Romania. The initial broad list of indicators comprised close to 50 potential measures across various domains of hospital QoC and patient safety. The list included indicators such as average time interval from patient registration to triage, mortality rates following total hip arthroplasties, artery bypass graft procedures and abdominal aortic aneurysms, diabetes lower extremity amputation rates, and hypertension hospital admission rates, to list a few. Using modified Delphi consensus-building method and involving multidisciplinary hospital teams and key stakeholders listed above, the indicators were ranked and selected according to their perceived meaningfulness, relevance, and feasibility, resulting in a final set of indicators to support the rollout of a national HQF [22–24]. Consensus-building exercises aimed to balance comprehensiveness with feasibility of data collection in the Romanian healthcare context. Meetings took place during three WHO country missions and one online consultation meeting with international expert in late 2022. More than 70 stakeholders, with roles and affiliations described in previous research steps, were involved during meetings that took place at the MoH, NHIH, ANMCS, CMR, OAMR and in seven hospitals (4 in Bucharest, 1 in Călărași, 1 in Cluj-Napoca and 1 in Iași) as well as in two primary care facilities. Finally, the research team developed methodological definitions, measurement workflows and inclusion criteria for each indicator using available literature.

### QoC indicator set piloting

In consultation with the Romanian MoH, a sample of public hospitals nationwide were selected to participate in a prospective 60-day pilot study, collecting data and reporting on the previously developed set of QoC indicators. Piloting aimed to evaluate the feasibility of data collection and suggest potential improvements for a nationwide rollout. The hospitals for the pilot study were selected using a convenience sample. The inclusion criterium was for participating hospitals to be public, managed and owned by the MoH and local authorities. Representativeness considered criteria of hospital categories and geographical areas. These criteria enabled working with hospitals from different contexts, including number of beds and care specialisation level (hospital categories) and various socioeconomic contexts (different geographical areas). Three Bucharest-based hospitals and three from other regions, with varied levels of specialisations and number of beds, were identified and joined the piloting. The selected hospitals represent a diverse range of healthcare facilities in Romania, including one university, two general and three specialised hospitals, with latter focusing on oncology, nephrology, and emergency care. Geographically distributed across different regions of Romania, with three located in Bucharest (the capital), and one each in Cluj-Napoca, Timisoara, and Călărași, they represent both major urban centres and a smaller city. These hospitals vary significantly in size and capacity, from large university and emergency hospitals with over 1000 beds and treating over 100.000 inpatients annually, to specialised institutes and county hospitals with approximately 300–700 beds serving 20.000–50.000 inpatients per year. A Ministerial Order was published in “Monitorul Oficial al României”, the official gazette of Romania, which formalised the list of participating hospitals, HQF, QoC indicator set and pilot project. The Ministerial Order mandated the collection and reporting of data for the set of QoC indicators and defined technical details, including rationale and calculation methodology for each. In preparation for the pilot study, the technical requirements for the indicator data collection IT platform were proposed, the IT solution provider was contracted, and the platform was developed and implemented in participating hospitals. The IT solution used for data collection and management was a secure, web-based platform developed specifically for this project. It featured a user-friendly interface for data entry, accessible to authorised hospital staff via secure login, built-in data validation rules to ensure data quality and consistency and basic ability to generate real-time reports and visualizations of indicator data. Additionally, the IT platform featured secure data storage compliant with Romanian and EU data protection regulations. WHO Regional Office for Europe also deployed several in-country missions to provide detailed on-site training and technical support for the six participating hospitals. Approximately 10–20 staff members were selected by hospital management to participate in the training in each participating hospital. This group typically included doctors, nurses, and hospital epidemiologists and statisticians. The selection process ensured representation from relevant departments such as intensive care units, operating rooms, general (internal medicine) wards, and emergency rooms, amongst others, aligning with indicators for which data was being collected. Trainings were organised in a full-day format. A 60-day prospective data collection pilot study ran in July and August, with the final data upload in September 2023—the analysis of submitted data used univariate statistics and focused on its completeness and coverage. [5, 9, 13].

Following indicator data collection and analysis, a full-day technical workshop was organised in Bucharest, Romania, in October 2023, to elicit participants’ feedback. Over 40 participants joined, mostly hospital managers and healthcare workers from piloting hospitals, national and international QoC experts, WHO representatives in Romania and the Romanian Minister of Health. During the workshop, a survey instrument, developed specifically for this study, was administered to participants with an aim of eliciting experiences working on the pilot project, focusing on the self-reported perceived enablers and barriers and the relevance of collecting and reporting specific data for the QoC indicator set. Verbal informed consent was obtained from all participants prior to their involvement in the survey. No personal identifying information or individual health data was collected or shared with the research team at any point during the study. Survey instrument questions are presented in the Supplementary materials. Pilot study results were presented and extensively discussed to contribute to the subsequent national rollout of the QoC indicator set. Participants contributed to group discussions on “Challenges and solutions during the pilot study” and “Readjusting indicators and methodologies”, moderated by authors working for the WHO. Group discussions were noted in detail, and the notes were inductively analysed using thematic analysis. This resulted in several critical insights and recommendations from pilot project participants and allowed for further development of a Technical Guidebook [25] to support the national adoption of these standard metrics.

### Lessons learned

To guide the next steps of the broader reform project, the research team distilled a set of lessons learned from the activities described earlier. In a modified nominal group technique approach, one author (DI) reviewed the data collected during the QoC indicator set development, piloting and post-piloting evaluation and proposed potential lessons learned, which were discussed and decided among all authors [26–28]. A systematic consensus approach was used to summarise expert opinions. As a result, the research team proposed several lessons learned to move forward in implementing the QoC indicator-based HQF in Romania. Additionally, it was envisioned that such insights could serve as a valuable resource for other healthcare systems across the WHO European Region and beyond in similar efforts.

## Results

### Priority domains and the set of QoC indicators for Romanian hospitals

Focusing on the quality and safety of care in the Romanian context, four main priority indicator domains were identified: (1) patient safety (including hospital-acquired infections), (2) patient experience, (3) healthcare workforce training and safety, and (4) care effectiveness. Starting with a broad list of potential indicators, a core set of 25 QoC indicators was finally developed. Each indicator was defined in detail, including the selection rationale and calculation methods covering QoC domains aligned with the abovementioned national needs and priorities. Table 2 presents the final set of 25 QoC indicators used in the subsequent piloting.

**Table 2:** Final set of 25 QoC indicators for public hospitals in Romania used in the piloting project

Domains	Indicators
Patient safety	Central line associated bloodstream infections rate
	Ventilator-associated events rate
	Percentage of in-hospital patients assessed for fall risk through applied protocols
	Incidence rate of patients’ falls during hospitalisation
	Percentage of in-hospital patients assessed for pressure ulcers’ risk through applied protocols
	Incidence rate of pressure ulcers acquired during hospitalisation
	Percentage of patients undergoing surgery where the Surgical Safety Checklist was applied
	Post-operative bleeding rate requiring surgical re-intervention
	Surgical site infections rate
Patient experience	Patient experience questionnaires’ completion rate
	Patient experience after hospital discharge rate
Healthcare workforce training and safety	Percentage of healthcare workers participating in training activities
	Percentage of healthcare workers that followed standard protocol for occupational health upon a sharp injury during working hours
	Percentage of healthcare workers with updated influenza vaccination schedule
Care effectiveness	In-hospital mortality by heart failure
	In-hospital mortality by acute myocardial infarction
	In-hospital mortality by pneumonia
	In-hospital mortality by ischaemic stroke
	Percentage of patients readmitted to the Intensive Care Units after 48 h after transfer
	Unscheduled readmission to hospital within 30 days of discharge for heart failure through the emergency room heart failure
	Average length of hospitalisation
	Percentage of surgeries with a perioperative hospital length of stay less than 48 h
	Pre-operative hospital length of stay
	Percentage of cancer patients whose nutritional status was assessed through applied protocols
	Time from admission to treatment for ischaemic stroke (door-to-needle time)

### The pilot study results and the post-piloting technical workshop insights and recommendations

**Fig. 1:**
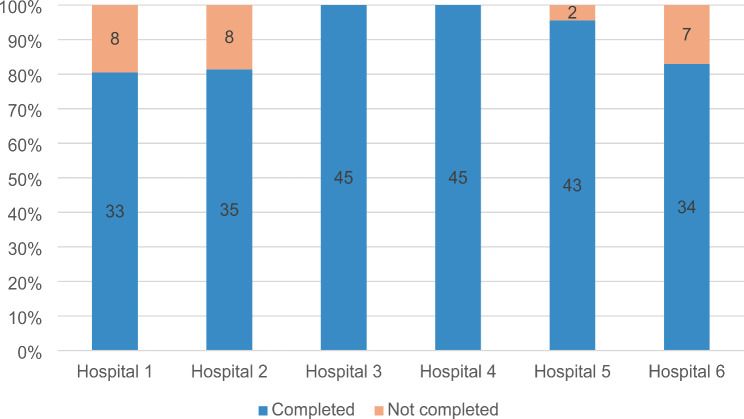
QoC indicator set data collection completion rate in piloting hospitals. Note: Showing data completeness for the 45 data points collected during the piloting study. This represents two reporting periods for most of the 25 indicators, with exceptions for the three “Healthcare workforce” domain indicators (collected once) and the “Patient experience after hospital discharge rates” indicator (not collected due to technical reasons). Additionally, please note that the maximum number of 45 data points did not apply to all participating hospitals, reflecting their narrow area of expertise. As an example, a specialised nephrology hospital did not report on indicators related to myocardial infarction or stroke, as these conditions are not routinely managed in such institution

Following a two-month pilot study that involved six hospitals of different profiles, data for over 80% of the applicable indicators was collected and analysed, as presented in Fig. 1.

Two hospitals reached full coverage of indicator collection and delivery of all 45 possible data points during the piloting period. Across all participating hospitals, the average completion rate was 90%, with data submitted for 235/260 possible indicator intervals across all six piloting hospitals.

Data collection involved both manual and automated processes. For patient safety domain indicators, more manual work was required, with data collected prospectively using standardised forms provided by the project and research team, as described in detail in the Technical Guidebook. This approach allowed for the capture of specific, detailed information not typically available in existing hospital datasets. In contrast, data for workforce and effectiveness domains’ indicators were largely extracted from existing statistical hospital datasets, requiring less manual intervention. This mixed approach ensured comprehensive and accurate data collection across all indicator categories.

A participants’ survey was conducted during the post-piloting technical workshop. It focused on the experiences and opinions of participants in the piloting project. Two-thirds of workshop participants (27/40) fully responded to the survey. On average, they found data collection for the QoC indicators in their hospitals to be relatively easy and helpful for the hospital’s activity. Specifically, the insights gained from collecting and analysing data for these indicators were used locally to identifying areas for improvement, inform decision-making, track progress and enhance staff engagement. The WHO training regarding the piloting process was rated as very useful. Participants also felt that their opinions and concerns were considered during the training and used to refine the definitions and methodologies of working with QoC indicators. All four indicator domains were assessed as relevant, considering the needs and priorities of the Romanian healthcare system and – in general - strengthening the QoC in Romania. Analysis of feedback from 27 post-piloting workshop participants involved in the piloting process revealed varying levels of difficulty in data collection across the 25 indicators. When asked to highlight “the three easiest indicators in terms of data collection”, participants listed “Average length of hospitalisation” (with 40.7% of respondents including them among “the three easiest indicators in terms of data collection”), “In-hospital mortality by acute myocardial infarction” (37.0%), and “In-hospital mortality by heart failure” (33.3%). Conversely, when asked about “the three hardest indicators in terms of data collection”, those considered most challenging were “Central line associated bloodstream infections rate” and “Ventilator-associated events rate” (both at 48.1% of respondents) and “Post-operative bleeding rate requiring surgical re-intervention” (25.9%). This variation highlights areas where additional support or refinement may be necessary in future implementations. Plans to implement QoC hospital indicators across the country were assessed as very beneficial for the patients. Following the piloting, most participants felt confident regarding their participation in the upcoming nationwide implementation of these QoC indicators. However, almost everyone considered that legislative changes are needed to ensure the proper implementation of these 25 indicators nationwide. The necessary legislative changes include establishing legal frameworks for inter-institutional data sharing, mandating hospital participation in quality improvement initiatives, and creating a legal basis for implementing a pay-for-performance system based on these quality indicators.

Workshop group discussions on “Challenges and solutions during the pilot study” and “Readjusting indicators and methodologies” resulted in several participants’ insights from the piloting phase and recommendations for the project’s continuation. The thematic analysis allowed the grouping of these insights and recommendations into several consideration areas, as presented in Table 3.

**Table 3:** Insights and recommendations collected from post-piloting workshop participants

Consideration areas	Insights and recommendations
Staff training	The engagement of healthcare professionals within hospitals was seen as a positive development, including working to set up teams for the QoC indicator data collection and reporting, enhanced teamwork and awareness of QoC and patient safety measurements and their use. The WHO training on indicators and the piloting process were very useful. Including more hospital staff in future staff training on working with indicators, both on data collection and on indicator data use for quality assurance and improvement.
Indicator methodologies	Data collection for the core QoC indicators was relatively easy and quite useful for participants’ hospital’s activity. Indicators in the “Healthcare workforce” dimension require further clarification and methodological development. For instance, due to organisational vaccination policies, it is not fully possible to report on the actual number of vaccinated healthcare workers. Data for the “Patient experience after hospital discharge rates” indicator was not collected during the piloting study for technical reasons. There is the need for a standardised tool to capture patient experience after hospital discharge. Different approaches in the determination of “patient days” caused inconsistencies in the calculation of indicators.
Data infrastructure	Some deficiencies in data infrastructure were observed, especially considering the paper-to-digital conversion of some data and the need to combine data from different data sources for a number of indicators. Review of the admission diagnosis is not currently possible in most hospital information systems. Updates to hospital information systems were needed to align with the measurement principles. Digitalisation strategy and implementation needs to complement work on indicators.
Hospital procedures	Focused teams were created for specific areas of measurement in some hospitals. Internal mechanisms should be adopted in order to sustain this process. Clinical governance structures (e.g., hospital committees) should take over the related responsibilities in order to make the program effective, efficient, and sustainable. In the development and application of the QoC indicators, internal auditing challenges were observed. Calculating indicators such as the “Percentage of in-hospital patients assessed for fall risk through applied protocols” and “Percentage of in-hospital patients assessed for pressure ulcers’ risk through applied protocols” required internal auditing, a practice not widely established across Romanian healthcare settings. This necessitated the development of bespoke procedures within each participating hospital, reflecting the hospitals’ size, patient capacity, and the availability of staff trained in internal auditing techniques.
New indicators	Suggestions were made to include the following indicators and indicator categories in future QoC indicator sets: medication safety-related incidents, patient identification-related aspects, blood and blood products management, hand hygiene, antibiotics use and antimicrobial resistance stewardship programmes, patient–doctor communication aspects and systematic psychological and mental evaluation of patients.

### Lessons learned

The authors identified several lessons learned through reflective and consensus-building approaches. Data collected during all of the above-described phases of the project was used. Lessons learned are presented in Table 4 below and discussed in detail in the following section.

**Table 4:** Lessons learned going forward for the Romanian healthcare system and other countries

Lessons learned
High-level commitment and organisational-level process ownership are crucial for quality assessment and improvement initiatives, such as this one.
Data and – equally importantly – data culture are prerequisites for working on QoC and patient safety.
All types and levels of interoperability are needed to proceed with data-supported QoC improvement work. These include: • Political interoperability - High-level political commitment; • Legal interoperability – Clear, supportive and protective legal framework; • Organisational interoperability – Aligned structures and processes, including improved clinical governance and internal auditing mechanisms; • Semantic interoperability – Defined and aligned data collection and reporting methodologies, including coding standards; and • Technical interoperability – Up-to-date data infrastructure to collect, report and exchange QoC indicators data.
Unless a no-blame and no-shame approach to quality assessment and improvement is introduced, the risk of unwanted consequences, including gaming, poses a threat to sustainable success of such initiatives.
Data and indicators are a means to an end—QoC and patient safety improvement—and not an end in itself.

## Discussion

This work developed and piloted a set of QoC indicators for public hospitals in Romania, with the aim of supporting the future work of a newly founded national pay-for-performance HQF. The ultimate goal of this work is to enhance the quality and safety of care provided in public hospitals in Romania and the health outcomes of individuals and populations. Through a multi-phase mixed-methods process, a set of 25 QoC indicators was developed, across all four priority domains, and piloted in six Romanian hospitals. The results revealed promising data collection and participation levels, with an average completion rate of 90% across all indicators in six piloting hospitals. Insights from the post-piloting workshop underscored the perceived ease and usefulness of data collection and the identified relevance of the indicators for improving healthcare quality. Notably, participants expressed confidence in the nationwide implementation of these indicators, albeit acknowledging the need for continued system- and organisation-level support, further developments in data infrastructure and governance, as well as the appropriate legislative changes to support the effective implementation of such efforts.

The main strength of this work lies in its contribution to the practice-based research corpus. This paper offers valuable, real-world insights into processes, methods and results of developing and piloting a new set of QoC indicators in public hospitals in Romania in 2022 and 2023. It also lists and discusses lessons learned for future quality improvement work in Romania and potentially in other national contexts. Several limitations should also be acknowledged. As only six out of 366 public hospitals in Romania were included in the pilot study, its representativeness is limited, and caution should be exercised in generalising findings [5]. Furthermore, the relatively small number of post-piloting workshop participants may have negatively influenced the depth and breadth of insights gathered. As described earlier, data for one QoC indicator in the set was not collected during piloting due to technical reasons. A final limitation is that, in a hospital-centric healthcare system, this work again mostly focuses on hospital-level QoC metrics. Despite this limitation and relying on data collected in hospitals, efforts were made to introduce certain proxy indicators beyond in-patient hospital care. An example is the surgical site infection rate indicator, with a one- to three-month follow-up period, providing a proxy of post-discharge care in a hospital outpatient setting but also on other healthcare system levels, including primary care. Using such indicators also provides insights into the status of the health information system in Romania, the level of its integration, but also on patient mobility patterns. Balancing what is ideally and practically achievable, trade-offs were made to collect available data and start developing a data culture focused on quality improvement [21, 29]. The development of these QoC indicators aims to lay the groundwork for future national benchmarking efforts. While this study focused on initial implementation and feasibility, future phases will address the critical aspects of data comparability across institutions. This process will involve continued refinement of indicator definitions, data collection methodologies, and reporting standards to ensure meaningful comparisons can be made, supporting the ultimate goal of driving quality improvement across the Romanian healthcare system. Finally, our approach acknowledges the multidisciplinary nature of healthcare delivery and shared responsibility across various healthcare professionals – including physicians and nurses – but also patients as well as system-level factors in influencing these outcomes. Future research and practice-oriented efforts should consider a broader health system perspective and service integration across levels, including, for instance, primary health care, mental care, and childhood and maternal health.

The piloted QoC indicator set does include Patient-Reported Experience Measures (PREMs) through the “Patient experience after hospital discharge rates” indicator. The inclusion of PREMs as a *placeholder* indicator, despite the current lack of implementation in Romanian hospitals, serves as a development metric and initial step towards more comprehensive patient-reported measures. This approach allows for a gradual integration of patient perspectives into quality assessment, considering the current capabilities of the Romanian healthcare system.

Several key lessons emerged from this study, highlighting critical enablers for successful and sustainable quality improvement initiatives. Firstly, the success of quality assessment and improvement initiatives, such as the one undertaken in this study, depends significantly on securing high-level commitment from political and organisational leadership [3, 30]. This commitment provides the necessary drive to implement change and allocate resources effectively. Otherwise, the sustainability of such initiatives is at risk due to insufficient support or shifting priorities. In addition to political endorsement, fostering process ownership within individual healthcare organisations is crucial. When healthcare facility-level stakeholders share ownership over quality improvement processes, they are more likely to engage actively, leading to sustained progress. Such ownership fosters a culture of accountability and empowerment, encouraging frontline staff to contribute ideas and take initiative in implementing quality improvement measures tailored to their specific contexts [17, 18, 21].

Furthermore, central to any QoC and patient safety improvement work are robust data systems and, equally importantly, a supportive data culture. Effective quality assessment and improvement rely on timely access to accurate and relevant data. This necessitates investments in data infrastructure, including interoperable electronic health records and data collection and validation mechanisms, to ensure comprehensive and reliable information availability [30–33]. Building a data culture within healthcare organisations – one where data-driven decision-making is integrated into everyday practices – is crucial. This involves providing staff with the necessary training and tools to collect and analyse data and fostering a culture where data is valued as a cornerstone of quality improvement efforts. A key aspect of fostering a data culture is the active involvement of frontline staff, including physicians and nurses, in the data collection and interpretation processes, which can significantly contribute to building data literacy and promoting a positive approach to using data for quality improvement rather than punitive purposes [34]. Organisations can encourage transparency and collective problem-solving by creating a safe, improvement-focused environment to drive meaningful quality improvements. Peer support and sharing experiences play a pivotal role in this process, facilitating the exchange of ideas and experiences [3, 33].

Achieving meaningful progress in data-supported quality improvement work requires addressing various dimensions of interoperability [35–37]. Political interoperability entails securing high-level political commitment to prioritise and support quality improvement initiatives. This commitment ensures that resources are allocated, policies are formulated, and stakeholders are mobilised effectively. Legal interoperability involves establishing a clear, supportive, and protective legal framework that enables the collection, sharing, and use of healthcare data while safeguarding patient privacy and confidentiality. Organisational interoperability requires aligning processes and fostering a data culture across healthcare organisations, ensuring seamless collaboration and information exchange. These include improved clinical governance, strategic approaches to health data infrastructure and its governance, and established internal auditing mechanisms. Semantic interoperability necessitates standardising data collection and reporting methodologies, including adopting coding standards, to facilitate data linkage, aggregation and analysis. Finally, technical interoperability relies on maintaining up-to-date data infrastructure capable of efficiently collecting, reporting, and exchanging QoC indicators data, including the context of identified patient mobility patterns.

Finally, while the phases described here—developing, piloting and implementing QoC indicators—are important, it is crucial to recognise that indicators are a tool that serves to ultimately improve patient outcomes and enhance the QoC. Therefore, while ongoing indicator monitoring, analysis, and refinement efforts are necessary, on their own, they are insufficient to drive continuous improvement and achieve the overarching goal of data-driven, people-centred care. Additionally, efforts should be made to complement, rather than substitute, existing accreditation processes, recognising the value of both approaches in promoting healthcare quality. Only when selected organisational and bedside interventions are implemented in parallel to system-level ones can such efforts result in sustainable improvement.

This work’s major policy, practice, and research implications lie in its systematic approach to describing and learning from a real-life quality improvement initiative in Romanian public hospitals. It lays the groundwork for further quality assessment and improvement efforts by defining and piloting a set of QoC indicators and meticulously describing the process. As such, it enables further implementation of the national HQF through its first funding phase, which will focus on generating data to explore and refine the further steps towards potential pay-for-performance models for hospitals in Romania. For their use, the actionability of these indicators is crucial, as is ensuring they are suitable and purposeful. This includes their technical feasibility, relevance, and alignment with national healthcare priorities. The evidence-informed and stakeholder-contextualised development and implementation of these indicators presents a crucial step towards sustainably enhancing healthcare quality in Romania [21].

While this study was conducted within the Romanian healthcare context, many aspects of our approach and findings may be applicable to other countries, particularly those in similar stages of healthcare system development. Romania-specific elements include the current structure of the healthcare system, existing data infrastructure, and specific cultural and organisational challenges. However, the methodology used for developing and piloting QoC indicators, the emphasis on stakeholder engagement throughout the process, and the phased approach to implementation could be adapted for use in various national contexts. Many of these QoC indicators, such as those related to mortality rates, and readmissions, are widely used internationally [3, 16, 30]. However, some indicators were tailored to address particular challenges in the Romanian healthcare system, such as those on HAIs. The selection of indicators reflects a balance between internationally recognised measures and those addressing Romania’s specific needs and priorities. The lessons learned regarding the importance of high-level commitment, fostering a data culture, and ensuring interoperability across multiple dimensions are likely to be relevant in many healthcare systems globally. Countries looking to implement similar quality improvement initiatives could benefit from our experiences while adapting the specifics to their local context.

## Conclusions

This work presents a significant development in supporting QoC and patient safety improvements in Romanian public hospitals. It does so by developing a set of QoC indicators and implementation methodologies and, through piloting, sheds light on such initiatives’ feasibility, effectiveness and future improvement opportunities. Key findings underscore the pivotal role of high-level commitment, stakeholder engagement, and a robust data culture in driving successful quality improvement initiatives. By fostering collaborative environments and transparent, no-blame assessment practices, healthcare systems and organisations can work on sustainable quality improvements. The relevance of this study extends beyond Romania, offering valuable insights for healthcare systems in the WHO European Region and globally.

## Electronic supplementary material

Below is the link to the electronic supplementary material.

Supplementary Material 1

## Data Availability

The datasets used and/or analysed during the current study are available from the corresponding author upon reasonable request.

## Abbreviations

QoC: quality of care
UHC: Universal Health Coverage
EU: European Union
GDP: Gross Domestic Product
HAIs: Healthcare-associated infections
EC: European Commission
RRP: Recovery and Resilience Plan
NHIH: National Health Insurance House
ANMCS: National Authority of Quality Management in Health (Autoritatea Naţională de Management al Calităţii în Sănătate)
CMR: Romanian College of Physicians (Colegiul Medicilor din România)
OAMR: Order of Nurses, Midwives and Medical Assistants in Romania (Ordinului Asistenţilor Medicali Generalişti, Moaşelor şi Asistenţilor Medicali din România)
OECD: Organisation for Economic Co-operation and Development
MoH: Ministry of Health
HQF: Health Quality Fund
WHO: World Health Organization
IT: Information technology
PREMs: Patient-Reported Experience Measures
