# Supplementary material for: Developing and piloting a set of quality-of-care indicators for Romanian public hospitals as part of a national programme to fund quality
Source: BMC Health Serv Res. 2024 Oct 16;24:1242. doi: 10.1186/s12913-024-11462-6 (PMC11481585; doi:10.1186/s12913-024-11462-6)
Supplement: Supplementary file 1 — Supplementary Material 1 [file 12913_2024_11462_MOESM1_ESM.pdf]

Supplementary file 1

“Post Pilot Study Survey”

This survey aims to capture the feedback from the healthcare professionals and managers of the hospitals enrolled in the Pilot Study for the Reform 1 of the Romanian NRRP (Quality of Care), conducted under the scope of the Ministerial Order number 2.567/2023, with the technical support of the World Health Organization (WHO).

The questions address individual opinions from the healthcare professionals and managers of the hospitals enrolled in the Pilot Study about the quality of care indicators established by the Ministerial Order number 2.567/2023. The survey is anonymous and is completed on a voluntary basis.

1.1.
How easy was the data collection for the 25 indicators in your hospital?
(1-10 Likert-scale scale responses, from “Not easy at all” to “Completely easy”)

1.2.
Which were the three easiest indicators in terms of data collection?
(A three-item selection across “Indicators 1-25”)

1.3.
Which were the three most difficult indicators in terms of data collection?
(A three-item selection across “Indicators 1-25”)

1.4
How relevant are these 25 indicators for your hospital activity?
(1-10 Likert-scale scale responses, from “Not relevant at all” to “Completely relevant”)

1.5.
Which are the three most important indicators to improve the quality of care in your hospital activity?
(A three-item selection across “Indicators 1-25”)

2.1.
How useful was the training provided by the WHO team for the Pilot Study?
(1-10 Likert-scale scale responses, from “Not useful at all” to “Very useful”)

2.2. How much did you feel your opinion and concerns were taken into account for refining the definition and methodology of these 25 indicators?
(1-10 Likert-scale scale responses, from “Not at all” to “Fully”)

2.3. How much did you feel your opinion and concerns were taken into account during the training for the Pilot Study?
(1-10 Likert-scale scale responses, from “Not at all” to “Fully”)

3.1.
How relevant are "Patient Safety" indicators considering the needs and priorities of the Romanian healthcare system?
(1-10 Likert-scale scale responses, from "Not relevant at all" to "Completely relevant")

3.2.
How relevant are "Patient Experience" indicators considering the needs and priorities of the Romanian healthcare system?
(1-10 Likert-scale scale responses, from "Not relevant at all" to "Completely relevant")

3.3.
How relevant are "Healthcare Workforce" indicators considering the needs and priorities of the Romanian healthcare system?
(1-10 Likert-scale scale responses, from "Not relevant at all" to "Completely relevant")

3.4.
How relevant are "Effectiveness" indicators considering the needs and priorities of the Romanian healthcare system?
(1-10 Likert-scale scale responses, from "Not relevant at all" to "Completely relevant")

3.5.
How relevant are these 25 indicators to strengthening the quality of care in Romania?
(1-10 Likert-scale scale responses, from "Not relevant at all" to "Completely relevant")

3.6.
How beneficial do you think the implementation of these 25 indicators across the country would be for the patients?
(1-10 Likert-scale scale responses, from "Not beneficial at all" to "Completely beneficial")

3.7.
How beneficial are these 25 indicators to improve the accreditation program for Romanian hospitals?
(1-10 Likert-scale scale responses, from "Not beneficial at all" to "Completely beneficial")

3.8.
Do you consider legislative changes are needed to ensure the proper implementation of these 25 indicators across the country?
(Single choice, Yes/No reply)

3.9.
After participating in the pilot study, how confident are you to be enrolled in the nationwide implementation of these 25 indicators?
(1-10 Likert-scale scale responses, from "Not confident at all" to "Completely confident")
